# Asymptomatic Coronary Spasm Due to Polytraumatism

**DOI:** 10.5935/abc.20170093

**Published:** 2017-08

**Authors:** Maria Cruz Aguilera, Jorge Restrepo, Fernando Rivero, Teresa Bastante, Rio Aguilar, Fernando Alfonso

**Affiliations:** Hospital Universitario de La Princesa, Madrid - Spain

**Keywords:** Coronary Vasospasm, Angina Pectoris, Endothelium/physiopatolhology, Wounds and Injuries

## Introduction

Variant angina is a form of angina pectoris caused by vasospasm of an epicardic coronary artery that results in myocardial ischemia that might be manifested by transient ST segment elevation on the electrocardiogram.^1^ Although it usually presents with chest pain, asymptomatic episodes are not rare.^2,3^ We report a case of an incidental diagnosis of asymptomatic coronary vasospam in a patient with polytrauma.

### Description

**Figure 1:**
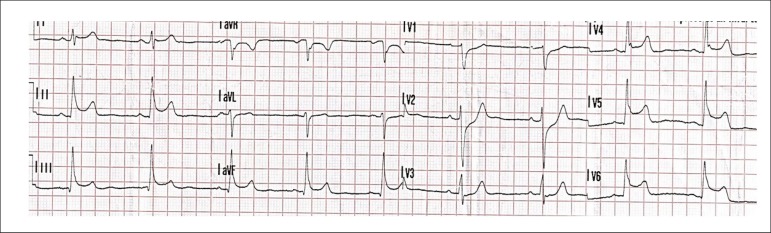
ST-segment elevation on first ECG performed by emergency medical services.

A 42-year-old man with a past medical history of smoking and no previous cardiovascular disease was admitted to the emergency department because of an accidental fall from 3 meters high with cranial, facial and thoracic traumatism. As part of the initial evaluation a 12-lead electrocardiogram was performed by emergency medical services showing a transient 3 mm ST-segment elevation in II, III, aVF, V5 and V6 leads which was not longer present in the first ECG performed at hospital admission (Figure 1). Patient denied any chest pain, dizziness or dyspnea prior to the accident nor after it. Physical examination, including neurological basic explorations, was completely unremarkable. Cranial, thoracic and abdominal computed tomography scans were performed without evidence of any organ damage. Blood analyses reported normal values of cardiac biomarkers. A transtorathic ecocardiogram showed a normal left ventricle size with preserved systolic function, without segmental wall motion abnormalities, and absence of pericardial effusion.

**Figure 2:**
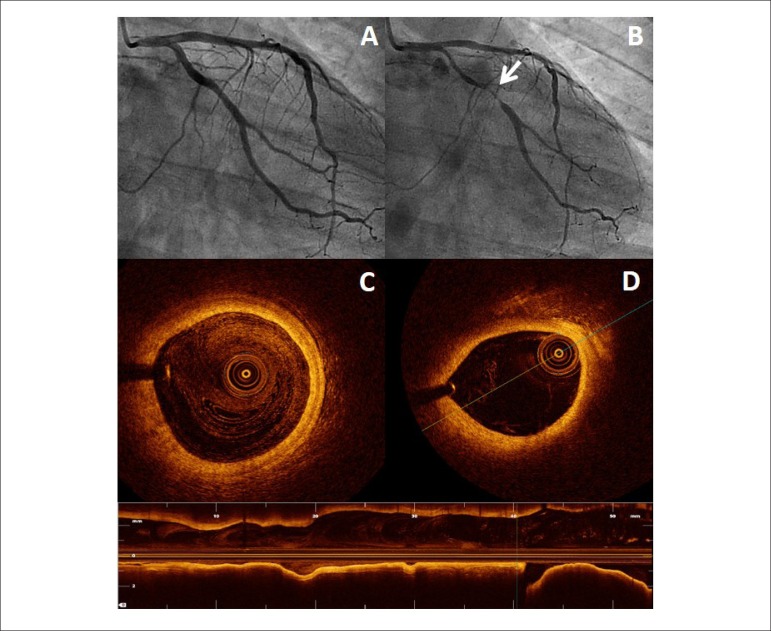
A) Basal coronary angiography with non-significant stenosis. B) Ergonovine-induced severe coronary vasospasm of the circunflex coronary artery (arrow) with a very mild diffuse calliber reduction of the entire coronary tree. C) Normal coronary wall structure. The characteristic 3-layered appearance is readily visualized. D) Uncomplicated atherosclerotic lipid plaque at the same coronary segment where vasospasm was induced.

The patient was admitted to the cardiology ward remaining completely asymptomatic with no changes in serial ECGs, troponin determinations or ecocardiographic explorations. However, due to the high suspicion of coronary vasospasm, with the presence of transient ischemic changes on ECG, catheterization was planned and performed 24 hours later. Coronary angiography revealed mild lumen irregularities, mainly on the left circumflex coronary artery, but no significant coronary stenosis. An ergonovine test was performed. The test was clinically and electrically positive, with weak chest pain and 3 mm ST-segment elevation in II, III and aVF leads. A severe vasospasm in proximal segment of circumflex artery was also documented (Figure 2). Intracoronary nitroglycerin was given leading to a complete resolution of the angiographic changes and ST-segment normalization. Optical coherence tomography revealed an uncomplicated lipid-rich plaque at the target segment. No data suggestive of minor plaque rupture or intracoronary thrombus was revealed.

With the final diagnosis of vasospastic angina, the patient was discharged four days later under treatment with calcium channel blockers and low-dose of aspirin. At three months follow the patient remained completely asymptomatic without new cardiovascular events.

## Discussion

This case meets the vasospasm guideline's criteria required for the diagnosis of "definite vasospastic angina".^4^ We observed transient ischemic changes on the ECG that were reproduced in the catheterization laboratory during the provocation test with clear drug-induced angiographic coronary spasm that resolved after vasodilators. Pathophysiology of this syndrome includes endothelial disfunction and increased oxidative stress.^5^ In addition, the important role of disbalance of autonomic nervous system has also been well defined in its development.^6^ Smoking is also a well-known risk factor for vasospasm. However, in the case we present here, cathecolamine discharge after trauma may have played an important role which can have led to sudden excessive coronary vasoconstriction. Interestingly, coronary vasospasm has also been reported with other unusual presentations like severe arrhythmias or even Tako-Tsubo syndrome.^7^ However, in our patient a completely normal left ventricular function was demonstrated. To the best of our knowledge, this is the first case of asymptomatic variant angina that is diagnosed incidentally as a consequence of other acute clinical entity that precipitates coronary vasospasm.

In addition, in our patient OCT was instrumental to rule out plaque rupture or intracoronary thrombus formation that have been described in patients with vasospastic angina. Prior studies have recently suggested that erosion of a fibrotic underlying plaque with superimposed white coronary thrombus can be identified in most patients with coronary vasospasm.^8-10^ In our case, however, subtle images of rupture, erosion or thrombus were ruled out.

Our findings highlight the wide spectrum of this unique pathology. A better understanding of the pathophysiology of this challenging clinical entity is warranted to better identify potential precipitant factors of coronary vasospasm and thus obtain an early diagnosis.
